# The Influence of Performance Feedback on Trust and Self-Confidence in Dynamically Reliable Automation

**DOI:** 10.1177/10711813251367370

**Published:** 2025-09-01

**Authors:** Christopher Holland, Heather F. Neyedli

**Affiliations:** 1 Department of Psychology and Neuroscience, Dalhousie University, Halifax, NS, Canada; 2 School of Health and Human Performance, Dalhousie University, Halifax, NS, Canada

**Keywords:** human-automation interaction, trust in automation, self-confidence, performance feedback, dynamic reliability, automation reliability

## Abstract

This study examines how performance feedback influences trust and self-confidence during interactions with dynamically reliable automation. Trust and self-confidence are crucial components of human-automation collaboration, governing reliance decisions and decision-making processes. In this experiment, 80 participants engaged with an automated assistant whose reliability fluctuated across tasks, receiving performance feedback throughout. Contrary to expectations, trust and self-confidence remained stable, showing little sensitivity to changes in reliability or feedback. This suggests that performance feedback may moderate variability in trust, stabilizing perceptions of automation over time. However, this stabilization could lead to complacency and overconfidence. To develop systems that promote calibrated trust and optimize team performance, future research should investigate individual differences in trust calibration, situational awareness, and prior experience with automation. Understanding the complex interplay between feedback, trust, and self-confidence is essential for effective human-automation collaboration in dynamic environments.

## Introduction

The integration of automation into various aspects of modern life has underscored the importance of understanding human-automation interactions (Merritt et al., 2015; Wiegmann et al., 2001). At the core of effective collaboration between humans and automated systems is the concept of trust. Trust in automation is not a static entity but rather a dynamic variable that evolves over time, influenced by numerous factors including the reliability of the automation itself (Hoff & Bashir, 2015; Lee & See, 2004; Williams et al., 2023). The dynamics of trust are complex, and its optimization is crucial for enhancing decision-making processes and overall system performance in human-automation collaborative efforts.

Beyond trust, self-confidence emerges as another critical factor influencing how individuals interact with automated systems. Performance feedback provides insight into both the automation’s reliability and the user’s own accuracy thereby playing a role in shaping both trust in the automated system and self-confidence (Bartlett & McCarley, 2019). However, the interaction between these elements, especially under conditions of dynamically changing automation reliability, remains an area of ongoing investigation (Rittenberg et al., 2024; Yang et al., 2023).

Previous research has highlighted that initial reliability levels can have a lasting impact on user trust, with trust sometimes decreasing over time despite continued reliance on the automation (Rittenberg et al., 2024). This phenomenon suggests a potential mismatch between perceived and actual performance, possibly due to users’ inability to calibrate their confidence accurately in relation to the automation’s capabilities given that in the study, participants were not provided feedback on the accuracy of their decisions. The inclusion of performance feedback could mitigate this issue by enabling users to adjust their trust levels more appropriately in response to changes in automation reliability.

Furthermore, the influence of feedback on self-confidence is less clear-cut. While feedback might initially impact self-confidence, its long-term effects and how it interacts with trust dynamics are not well understood. This knowledge gap is significant because understanding how feedback moderates trust and self-confidence is essential for designing more effective human-automation interfaces that fosters appropriate reliance and optimal human-machine performance.

This study aims to contribute to the evolving understanding of human-automation interactions by exploring the impact of performance feedback on trust and self-confidence in scenarios where automation reliability varies. By examining how individuals adjust their trust levels and self-confidence in response to feedback under different conditions of automation reliability, we can gain valuable insights into the complex dynamics at play. Ultimately, this research seeks to inform the development of more sophisticated and adaptive automated systems that can better support human decision-making and performance, thereby enhancing the safety and efficacy of human-automation collaborations across various domains.

### Hypotheses

In this experiment where we provide feedback to participants, it is expected that;

1. Trust in automation will follow changes in the reliability of the automation (i.e., when reliability goes up, trust goes up, and vice versa)
2. Self-confidence will change in early blocks as participants calibrate their confidence to their actual performance and learn the task, but will level out in later interactions

## Method

This study employed an experimental design to investigate the impact of automation reliability on trust and self-confidence. A total of 80 participants were recruited through Amazon Mechanical Turk and compensated based on their accuracy during the task to motivate performance.

### Task and Procedure

**Figure 1.**
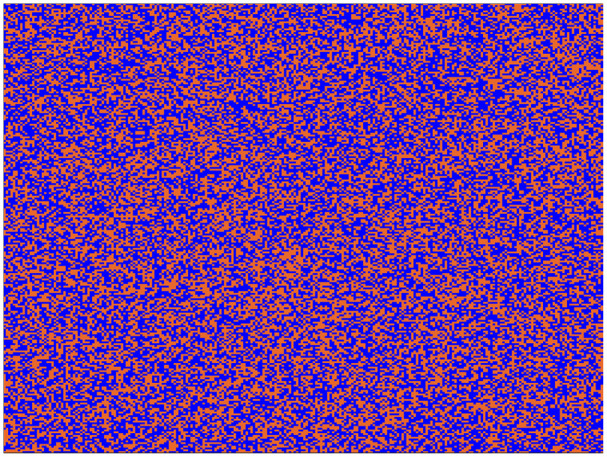
Example stimulus used for trials.

Participants completed a binary color decision task, where they had to determine if an image was mostly composed of orange or blue pixels (51:49 ratio), similar to the stimulus used by Bartlett and McCarley (2019) and methods in Rittenberg et al. (2024; see Figure 1). The task consisted of a series of trials, where participants viewed an image, followed by a recommendation from an automated assistant. Participants then had the opportunity to agree or disagree with the automation’s recommendation. Critically, after each trial, participants received feedback on their accuracy and the true answer, allowing them to evaluate the automation’s performance and their own decision-making.

### Automation Reliability Manipulation

Participants were randomly assigned to one of two groups: increasing or decreasing automation reliability. The automation reliability changed in 10% increments across blocks, with six blocks in total (50%, 60%, 70%, 80%, 90%, and 100%, or the reverse order for the decreasing automation group). Each block consisted of 50 trials, resulting in a total of 300 trials per participant.

### Trust and Self-Confidence Measures

**Figure 2.**
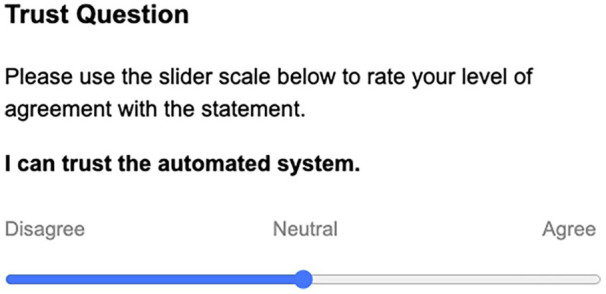
Trust question VAS, presented every 10 trials.

**Figure 3.**
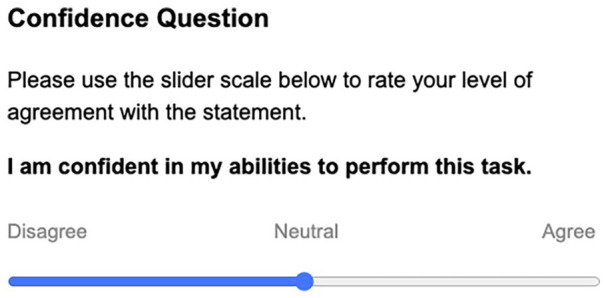
Self-confidence question VAS, presented immediately after trust question.

Trust in automation and self-confidence were measured using visual analog scales (VAS) at regular intervals throughout the experiment (Figures 2 and 3). For trust participants responded to the statement “I can trust the automated system” and for confidence “I am confident in my abilities to perform the task” with the VAS showing the anchors Disagree [at the far left]; Neutral [middle] and Agree [at the far right side]. Participants completed the VAS every 10 trials, resulting in a total of 30 measurements per participant. The VAS allowed participants to report their current level of trust in the automation and confidence in their own decision-making abilities.

### Design and Analysis

The experiment employed a between-subjects design, with participants randomly assigned to either the increasing or decreasing automation reliability group. The effects of automation reliability on trust and self-confidence were examined using repeated-measures analyses, taking into account the within-subjects factor of trial, block and the between-subjects factor of automation reliability group. This design allowed us to investigate how changes in automation reliability influence trust and self-confidence over time, while controlling for individual differences in participant performance.

## Results

### Trust

There was a main effect of group with participants initially experiencing reliable automation displaying higher trust throughout the study (*F*(1,452) = 28.131, *p* < .001). However, there was no effect of block or interaction between block and group indicating trust remained consistent in both groups across the experiment.

### Self-confidence

There was no effect of group, or block on self-confidence. Notably, self-confidence levels were similar between the two conditions

### Performance

**Figure 4.**
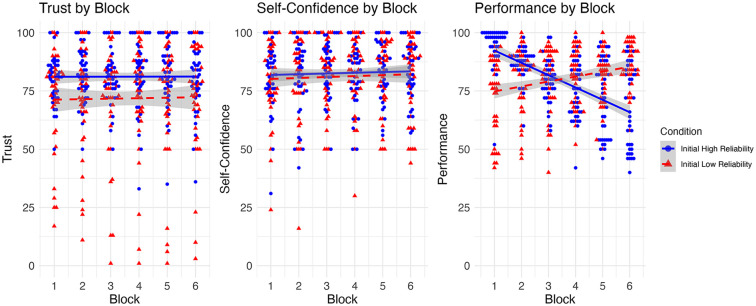
Trust, self-confidence, and performance by block. Trust and confidence were measured using For Peer Review visual analog scales, while performance is measured as a percent of the total number of correct responses for a given block.

Performance metrics revealed significant differences corresponding to an automation reliability and condition interaction (*F*(1,452) = 20.482, *p* < .001). For both conditions, performance followed the reliability of the automation (i.e., when automation was more reliable, participant performance was higher). However, there were differences in the strength of that relationship between conditions, with the decreasing reliability condition showing a stronger relationship between performance and automation reliability (Figure 4).

## Discussion

The aim of the present study was to examine how trust, self-confidence and performance changed when participants were provided with immediate feedback on their performance aided by automation. The initial reliability of the automation impacted trust in the automation, but trust did not change nor follow the automation reliability as it changed across the experimental blocks (hypothesis 1). Previous work has shown that the initial reliability level impacts trust in the system (Rittenberg et al., 2024), but in our experiment, trust decreased in both groups. In the previous experiments however, participants were not provided feedback on task accuracy so it may have been difficult to calibrate trust to the automation’s (and their own) performance. Therefore, performance feedback would appear to have stabilized trust dynamics. However, given that trust still did not change with changing reliability level, feedback alone does not appear to be enough to calibrate trust to automation reliability.

Even though trust did not change with reliability level, performance did change with automation reliability level suggesting that participants were relying on the automation throughout the experiment indicating a complacency effect (see also Holland et al., 2024, who directly measured dependence on the automation). Participants exposed to initially reliable automation may make an initial assessment that the automation is performing well, thus are less critical or aware of the automation performance in later trials, leading to over-reliance on automation. This over-reliance may result in a false sense of confidence, where participants trust the automation’s recommendations without critically evaluating its performance over time. This is particularly apparent because participants who started with more reliable automation showed a more drastic change in performance with changing reliability level than the group who started with initially less reliable automation. This finding indicates that along with lower trust level, the group who started with less reliable automation may have relied on the automated system less throughout the experiment. Taken together, it appears that initial reliability impacts trust and reliance behavior but changes in reliability level have less of an impact on these factors, even when participants have direct feedback of their performance.

Furthermore, the lack of changes in self-confidence levels (hypothesis 2) suggests that while feedback impacts trust, its influence on self-confidence may be more subtle or indirect. Participants may retain consistent self-confidence, relying on automation’s perceived reliability rather than personal judgments, unless more explicit cues challenge their self-assessments.

### Future Directions

A key area for future investigation involves examining individual differences in participants’ ability to detect changes in automation reliability and their skill at recalibrating trust levels accordingly. Identifying traits or cognitive processes that enhance an individual’s capacity to adapt trust in line with automation performance could inform the development of tailored feedback mechanisms. Additionally, exploring factors such as situational awareness, previous experiences with automation, or inherent skepticism may shed light on the determinants of effective trust calibration.

To achieve a comprehensive understanding of these dynamics, our future analyses will make use of more sophisticated models capable of capturing high-level interactions between trust, self-confidence, feedback, and reliance on the automation. This approach will enable us to better understand the complex network of factors shaping human behavior in human-automation interactions.

## Conclusion

Trust in automation did not change with changing reliability level when participants were provided performance feedback in the form of task accuracy, nor did it lead to participants shifting their self-confidence with experience in the task. It appears that initial trust may impact how participants were relying on the automation as measured through task performance. Future work and analyses are needed to understand the factors that could influence dynamic change in trust in automation with changing reliability level. Understanding these dynamics is essential for enhancing human-automation interactions and ensuring optimal performance.
